# RASGRP2 is a potential immune-related biomarker and regulates mitochondrial-dependent apoptosis in lung adenocarcinoma

**DOI:** 10.3389/fimmu.2023.1100231

**Published:** 2023-02-03

**Authors:** Yongting Liu, Yanhong Ouyang, Ziyang Feng, Zhaohui Jiang, Jiayao Ma, Xin Zhou, Changjing Cai, Ying Han, Shan Zeng, Shanshan Liu, Hong Shen

**Affiliations:** ^1^ Department of Oncology, Xiangya Hospital, Central South University, Changsha, Hunan, China; ^2^ National Clinical Research Center for Geriatric Disorders, Xiangya Hospital, Central South University, Changsha, Hunan, China; ^3^ Department of Emergency, Hainan General Hospital, Hainan Affiliated Hospital of Hainan Medical University, Haikou, Hainan, China; ^4^ Department of Radiotherapy, Tianjin First Central Hospital, Tianjin, China

**Keywords:** LUAD, RASGRP2, immune biomarkers, TF-miRNA-mRNA regulatory network, mitochondrial-dependent apoptosis

## Abstract

**Background:**

Ras guanine nucleotide-releasing protein 2 (RASGRP2), one of the guanine nucleotide exchange factors (GEFs), has attracted much attention in recent years. However, the correlation between RASGRP2 and immune infiltration and malignant features in lung adenocarcinoma (LUAD) has rarely been mentioned.

**Methods:**

The Limma package and the LASSO regression model were performed to screen for differentially expressed genes. Data from the TCGA and 5 GEO databases were used to explore the expression level of RASGRP2 in LUAD patients. A weighted co-expression network and LinkFinder module were established to find the related genes of RASGRP2. The ESTIMATE algorithm was used to analyze the correlation between RASGRP2 and immune infiltration in LUAD. Tumor-infiltrating immune cells were sorted and sequenced at the single-cell level to analyze differences in RASGRP2. Real-time PCR and immunohistochemistry were performed in the real-world cohort to verify the expression of RASGRP2 and its correlation with immune-related genes. Clone formation and EdU assays were used to verify the proliferation ability. The proportion of apoptotic cells was analyzed by flow cytometry. Observation of mitochondrial membrane potential (MMP) changes by fluorescence microscopy.

**Results:**

Our results suggested that decreased RASGRP2 was associated with worse clinical parameters and prognosis in LUAD patients. And we constructed a FLI1-HSA-miR-1976-RASGRP2 transcriptional network to support the role of RASGRP2. Enrichment analysis revealed that RASGRP2 was involved in lymphocyte activation and leukocyte adhesion. RASGRP2 was found to be positively correlated with the infiltration of most immune cells, immunoregulators, and chemokines in a subsequent study. Meanwhile, the real-world cohort confirmed that the expression levels of PDCD1, CTLA4, CD40LG, CCL14, CXCR5, and CCR7 were higher in the high-RASGRP2 expression group. Cytological experiments proved that RASGRP2 inhibited cell proliferation in LUAD by regulating mitochondrial-dependent apoptosis.

**Conclusion:**

RASGRP2 was a potential immune-related biomarker of LUAD. In addition, RASGRP2 was involved in the malignant progression of LUAD through the regulation of mitochondrial-dependent apoptosis.

## 1 Background

Lung adenocarcinoma (LUAD) is the major histological type of non-small cell lung cancer (NSCLC) that accounts for approximately 80% of lung cancer (1). LUAD occurs in distal airways and is associated with smoking and exposure to occupational and environmental pollution (2). Many therapeutic methods for LUAD, including surgery, chemotherapy, radiation, targeted therapy, immunotherapy, or comprehensive treatment have been approved (3–5).

In the past decades, many driver genes to cancers were found *via* genomic studies. TP53, KRAS, KEAP1, STK11, and EGFR are the most prevalent alteration genes in LUAD (3). The targeted therapies for several alteration genes have made big progress, such as EGFR-TKI (gefitinib, erlotinib, afatinib, and osimertinib) (6), and other targeted therapeutic agents, which target ALK (crizotinib, ceritinib, alectinib, brigatinib, andlorlatinib) (7), ROS1 (crizotinib, ceritinib, andlorlatinib), RET (cabozantinib) or MET (crizotinib) (8). Both preclinical and clinical therapeutic entities suggest that lung cancer treatment is going to be even more biomarker-driven (9). RAS proteins are known to be involved in cell signal transduction, cell proliferation, and differentiation in a variety of cancers, including LUAD (10). As we all know, guanine nucleotide exchange factors (GEFs) catalyze the conversion of GDP into GTP, thus activating the RAS protein (11). RAS proteins were once considered undruggable, so targeting GEFs may be a viable bypass (12, 13).

RASGRP2 gene encodes the Ca^2+^ and DAG-regulated guanine nucleotide exchange factor I (CalDAG-GEFI) (14). In this study, we explored the relationship between RASGRP2 and LUAD *via* bioinformatics analysis, lab experiments, and real-world cohort validation. Our results revealed that RASGRP2 was not only a novel biomarker involved in the malignant progression of LUAD but also influenced the tumor immune microenvironment (TIM), which might be useful for future cancer therapy.

## 2 Materials and methods

### 2.1 Public database

The clinical information and gene expression profiles of LUAD patients were obtained from TCGA database (RRID: SCR_014514) (https://portal.gdc.cancer.gov/). And we collected 5 datasets (GSE7670, GSE10072, GSE32867, GSE75037, and GSE116959) from the GEO database (RRID: SCR_005012) (http://www.ncbi.nlm.nih.gov/geo). After pre-processing for background correction, normalization and expression calculation, the probes are replaced with their official gene symbols according to the annotation file of the corresponding platforms. Any LUAD samples with incomplete clinical information were excluded.

### 2.2 Analysis of differentially expressed genes

DEGs were screened using the limma packages of R software in the 2 GEO database of LUAD (|log2(FC)| > 1, adj. P < 0.05). GEFs were obtained from GeneCards (RRID: SCR_002773) (https://www.genecards.org/). 40 GEFs were chosen for the next LASSO analysis. LASSO regression model was performed using the glmnet R package in TCGA-LUAD.

### 2.3 Comparison of the RASGRP2 expression level

The TCGA and GEO datasets were used to analyze the mRNA expression of RASGRP2 in LUAD. The CPTAC Data Portal (https://cptac-data-portal.georgetown.edu/) and Human Protein Atlas (HPA, RRID: SCR_006710) database (http://www.proteinatlas.org/) were used to verify the expression of RASGRP2 in LUAD at the protein level. T stage and pathological stage are defined following National Comprehensive Cancer Network (NCCN, RRID: SCR_012959) Guidelines (2022) (https://www.nccn.org/).

### 2.4 Survival and prognostic analysis

The survival package and survminer package were used to estimate the correlation between RASGRP2 expression and the survival rate of different clinical features in TCGA_LUAD patients. PROC package was used to establish the ROC curve of diagnosis.

### 2.5 RASGRP2 differential expression analysis

TCGA and GEO data were collected to analyze the mRNA expression level of RASGRP2 in LUAD patients and normal lung tissue samples. According to the mRNA expression level in TCGA, the top 25% are regarded as the high-expression group and the others are regarded as the low-expression group. We estimated RASGRP2 diagnostic and prognostic efficacy by using the ROC curve and Kaplan-Meier curve.

### 2.6 Weighted gene co-expression network analysis

We used WGCNA analysis to identify the important modules which have co-expressed genes with RasgGRP2 and the connection with the phenotype of interest and the core genes in the most important module. Based on the highest MS, we chose the key module to explore the hub gene to analyze the hub genes in the most significant module, we define the genes with higher MM and GS as the hub gene. In this study, |GS| > 0.5 and |MM| > 0.5 were chosen as the criteria.

### 2.7 LinkedOmics database analysis

The LinkedOmics database (http://www.linkedomics.org/login.php) is a multi-omics database that included 32 cancer types and their clinical information. It was chosen to identify the differentially expressed genes between LUAD and normal lung tissue in the TCGA data. The constructed co-expression network was shown in a volcano plot. 4803 genes (red spots) were positively correlated with RASGRP2, as well as 1658 genes (green spots) were negatively correlated (FDR < 0.05). A heatmap shows genes that are significantly positively or negatively correlated with RASGRP2 expression and its prognostic value. GO and KEGG were used to analyze the signal pathways in that co-expressed genes may participate.

### 2.8 TF-miRNA -mRNA regulatory network

Transcription factors (TFs) targeting RASGRP2 were predicted based on CHEA (https://maayanlab.cloud/Harmonizome/) and GRNdb (http://www.grndb.com/). miRNAs targeting RASGRP2 were predicted based on 3 different databases: miRWalk (RRID: SCR_016509) (http://mirwalk.umm.uni-heidelberg.de/), TargetScan (https://www.targetscan.org/vert_80/) and mirDIP (RRID: SCR_016770) (http://ophid.utoronto.ca/mirDIP/). StarBase V2.0 (RRID: SCR_016303) (https://starbase.sysu.edu.cn/index.php) was used to predict miRNA expression levels, prognostic value, and interaction sites. The overlapping result was pictured by the VennDiagram package (RRID: SCR_002414).

### 2.9 Specimen collection

Tissue samples were obtained from Xiangya Hospital (**Table S1**). Only the tissues diagnosed as LUAD and their adjacent tissues were included. Randomization and blinding are not applicable in this study. Samples were placed in sterilized cryogenic vials and quickly transferred to -80°C for storage. Formalin-fixed tissue samples were embedded with paraffin for immunohistochemistry (IHC) analysis. The studies were approved by the Ethics Committees of Xiangya Hospital. All participants/patients have given informed consent.

### 2.10 Cell culture

Human LUAD cell lines were obtained from the Cell Bank of Type Culture Collection of the Chinese Academy of Sciences. All cell lines were all maintained in RPMI-1640 (Gibco, USA) containing 10% FBS (Biological Industries, Israel) in a humidified incubator with 5% CO_2_ at 37°C. All cell lines were tested negative for mycoplasma contamination.

### 2.11 RNA isolation and quantitative real-time PCR

Total RNA was extracted using Trizol reagent (Invitrogen, USA) based on the manufacturer’s protocol. Quantification of RNA concentration using NanoDrop™ One.

1.5 µg RNA was transferred to cDNA using Evo M-MLV RT Kit (AG, China). The SYBR Green PCR Mastermix Kit (AG, China) was used to perform PCR amplification on QuantStudio™ Real-Time PCR software. 2^-ΔΔCt^ value was calculated to perform the quantitative analysis. The primers are as follows:

RASGRP2 forward: 5′-TGCTCCACATCTACCAACAATC-3′, reverse: 5′-GACGCTGTCTATGTCGATTAGG-3′;
GAPDH forward: 5′-GACCTGACCTGCCGTCTAGAAA-3′, reverse: 5′- CCTGCTTCACCACCTTCTTGA-3′;
CTLA-4 forward: 5′-GAAGTCTGTGCGGCAACCTA-3′, reverse: 5′- TGGGCACGGTTCTGGATCAAT -3′;
PDCD1 forward: 5′-GCTGCACTAATTGTCTATTGGG-3′, reverse: 5′- CACAGTAATTCGCTTGTAGTCG-3′;
CD40LG forward: 5′-GAGCAACAACTTGGTAACCCT-3′, reverse: 5′- GGCTGGCTATAAATGGAGCTTG-3′;
TNFRSF13B forward: 5′- GAGCAAGGCAAGTTCTATGACC-3′, reverse: 5′- CCTTCCCGAGTTGTCTGAATTG-3′;
CCL14 forward: 5′-CCAAGCCCGGAATTGTCTTCA-3′, reverse: 5′- GGGTTGGTACAGACGGAATGG-3′;
CCL19 forward: 5′- CTGCTGGTTCTCTGGACTTCC-3′, reverse: 5′- AGGGATGGGTTTCTGGGTCA-3′;
CXCR5 forward: 5′-CACGTTGCACCTTCTCCCAA-3′, reverse: 5′- GGAATCCCGCCACATGGTAG-3′;
CCR7 forward: 5′- ATTTGTTTCGTGGGCCTACTG-3′, reverse: 5′- TCATGGTCTTGAGCCTCTTGA -3′;

GAPDH was used as an internal control.

### 2.12 Immunohistochemistry

LUAD tissues were fixed with 4% paraformaldehyde, dehydrated, paraffin-embedded, and prepared into tissue chips. After dewaxing and hydration, 10 mM sodium citrate antigen repair solution was used at 95°C for 15 min for antigen repair, and then endogenous peroxidase was blocked by 3% H2O2 for 30 min at room temperature. Nonspecific antigens were blocked with 5% BSA in PBS for 30 min. The diluted RASGRP2 antibodies (GeneTex Cat# GTX108616, RRID: AB_1951644) were incubated overnight at 4°C. Next, antibody binding was detected with biotin-labeled IgG (Abcam Cat# ab6721, RRID: AB_955447). DAB and hematoxylin were then used for staining. Images were photographed with a microscope.

### 2.13 Gene overexpression and silencing experiments

To generate stable RASGRP2 overexpression cell lines, we purchased ZsGreen1-cMYC/pLVX-Puromycin (RRID: Addgene_180278) targeted RASGRP2 from HanBio. Multiplicities of infection (MOI) = 30. **The calculation of MOI was MOI = (lentivirus titer** × **lentivirus volume)/cell number.** The stable cell lines were screened using puromycin (Solarbio, Beijing, China) for 4 weeks and used for subsequent experiments. 3 si-RASGRP2 (si#1, 2, 3) and normal control (NC) were commercially obtained from Sangon (China). used Lipofectamine 3000 (Invitrogen, USA) was used to transfect. The siRNA sequences were used as follows: NC (5′-UUCUCCGAACGUGUCACGUTT-3′), si#1 (5′-CGCAAGAUGUCCCUGUUGUUUTT-3′), si#2 (5′-CUGCUCCACAUCUACCAACAATT-3′), si#3 (5′-GAUCAGUAUCAGACGGAGGAUTT-3′).

### 2.14 Immunofluorescence assays

The cells were seeded with 24-well plates. After fixing (4% paraformaldehyde, 15 min, room temperature) and blocking (3% BSA, 30 min, room temperature), the primary antibody (RASGRP2, 1:400, Mouse; PDL1, 1:200, Rabbit) was incubated at 4°C overnight. Then the corresponding fluorescent secondary antibody was incubated for 1 h at room temperature. Anti-fade 4′,6-diamidino2-phenylindole (DAPI) was used to label cell nuclei. Images were obtained using a fluorescence microscope.

### 2.15 Clone formation assay and EdU assay

For the clone formation assay, 1000 cells were seeded in 6-well plates/well, and the medium was changed every two days. After approximately 2 weeks, when cell colonies were visible to the naked eye, the medium was discarded and fixed with 4% paraformaldehyde. After that, the cells were stained with crystal violet solution for 30 minutes and rinsed 3 times with PBS. Take pictures to count the number of cell colonies. For the EdU assay, cells were seeded in 96-well plates and incubated with 50 µM EdU (C10310-1, RiboBio, China) for 2 hours for labeling. After the cells were fixed, 100 µL Apollo reagent per well was incubated for 30 minutes at room temperature in the dark. Finally, nuclear staining was performed. The number of Edu-positive cells was observed under a fluorescence microscope.

### 2.16 Apoptosis analysis

Annexin V-FITC Apoptosis Detection Kit was purchased from Beyotime. Adherent cells and cells in the medium were collected, and 195 μl Annexin V-FITC binding solution was added to gently resuspend, 5 μl Annexin V-FITC and 10 μl PI (propidium iodide) solution were added, and the mixture was gently mixed. Next, incubate at room temperature for 15 minutes in the dark. Flow cytometry (BD FACSCanto™II System, USA) was used to detect the proportion of apoptotic cells.

### 2.17 Measurement of mitochondrial membrane potential

The mitochondrial membrane potential was assessed using a Mitochondrial Membrane Potential Assay Kit with JC‐1 (C2006, Beyotime, China) according to the manufacturer’s instructions. JC-1 working solution was added to the cell culture medium and incubated at 37°C for 20 minutes. The fluorescence signals of the JC-1 aggregates (red) and monomers (green) were measured on the fluorescence microscope. The MMP levels were calculated as the red/green fluorescence ratio.

### 2.18 Statistical analysis

The differences among groups were detected with t-test. The survival analyses were determined by the Kaplan-Meier curve, log-rank test, and Cox proportional hazard regression mode. The correlation analysis was evaluated using spearman’s test. In all analyses, P-value < 0.05 indicated statistical significance, *, **, and *** indicate P < 0.05, P < 0.01 and P < 0.001, respectively.

## 3 Results

### 3.1 Screening of the overlap of DEGs and GEFs in LUAD

**Figure 1:**
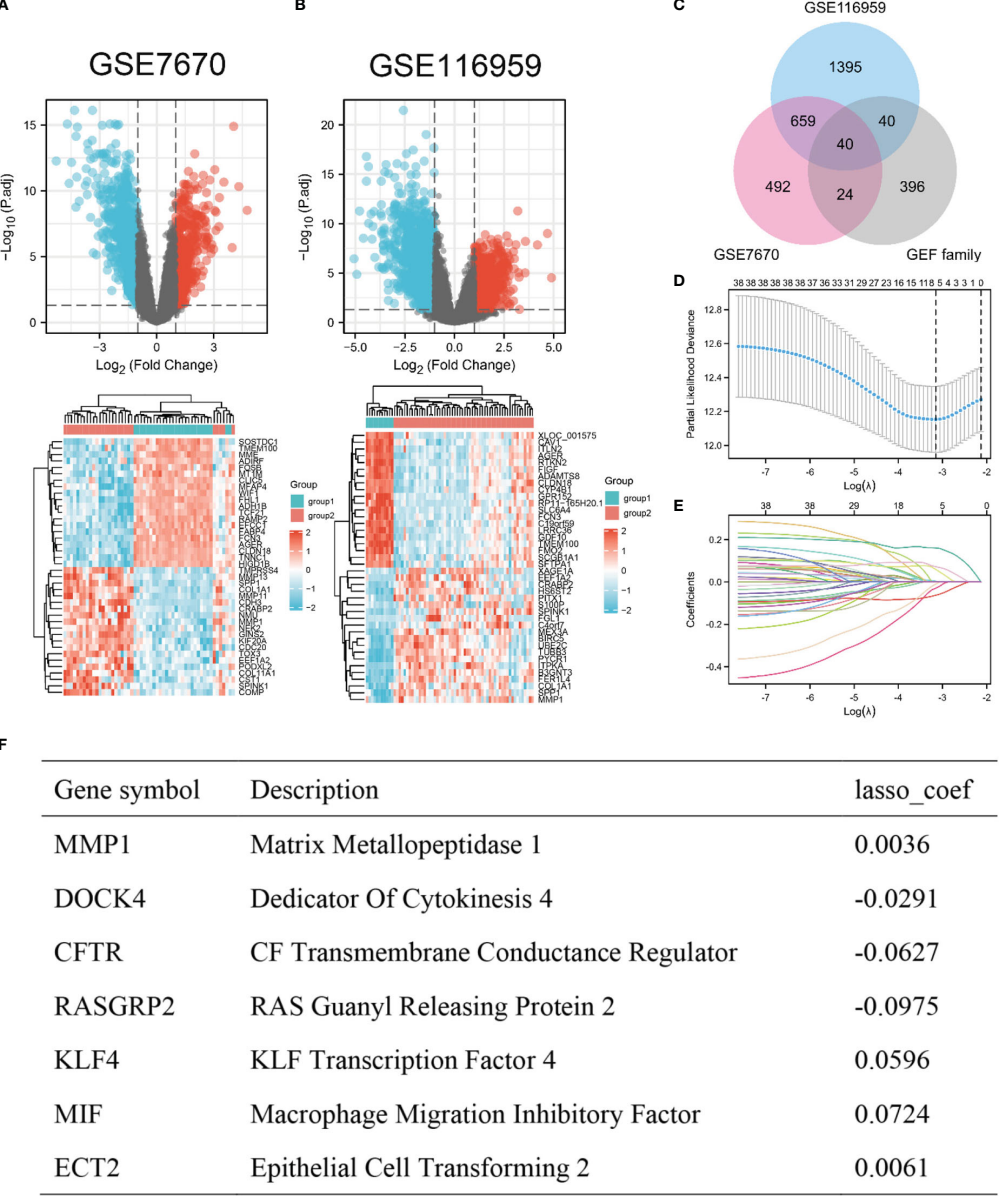
Screening of the overlap of DEGs and GEFs in LUAD. **(A, B)** Volcano plot (|log2(FC)| > 1, adj. P< 0.05) and heatmap plot (top 20) of DEGs in GSE7670 and GSE116959. **(C)** Overlap of DEGs and GEFs. **(D)** Cross-validation for tuning parameter screening in the LASSO regression model. **(E)** Coefficient profiles in the LASSO regression model. **(F)** Coefficients of 7 predictive genes in LASSO regression model.

A total of 715 up-regulated genes and 1419 down-regulated genes (|log2(FC)| > 1, P< 0.05) were obtained in GSE116959. A total of 493 up-regulated genes and 722 down-regulated genes (|log2(FC)| > 1, P< 0.05) were obtained in GSE7670 (**Figures 1A, B**). Due to a large number of differential genes, only the top 20 up-regulated and down-regulated genes were shown here as heatmaps, respectively (**Figures 1A, B**). According to the relevance score, the top 500 GEFs were extracted from the GeneCards (**Table S2**). We screened 40 interested genes by overlapping 2134 DEGs in GSE116959, 1215 DEGs in GSE7670, and 500 GEFs (**Figure 1C**). Subsequently, the LASSO regression algorithm was used to refine the gene sets by calculating regression coefficients (**Figures 1D, E**). In this manner, 7 proteins were identified as the most valuable GEFs (**Figure 1F**). The clinical correlation analysis revealed that only RASGRP2 expression levels were statistically significant in different T and pathologic stages of LUAD (**Figures S1A, B**). And the role of RASGRP2 in LUAD has not been systematically reported. Therefore, RASGRP2 was selected for the follow-up mechanism exploration.

### 3.2 Analysis of the expression of RASGRP2 and its prognostic and diagnostic value in LUAD

**Figure 2:**
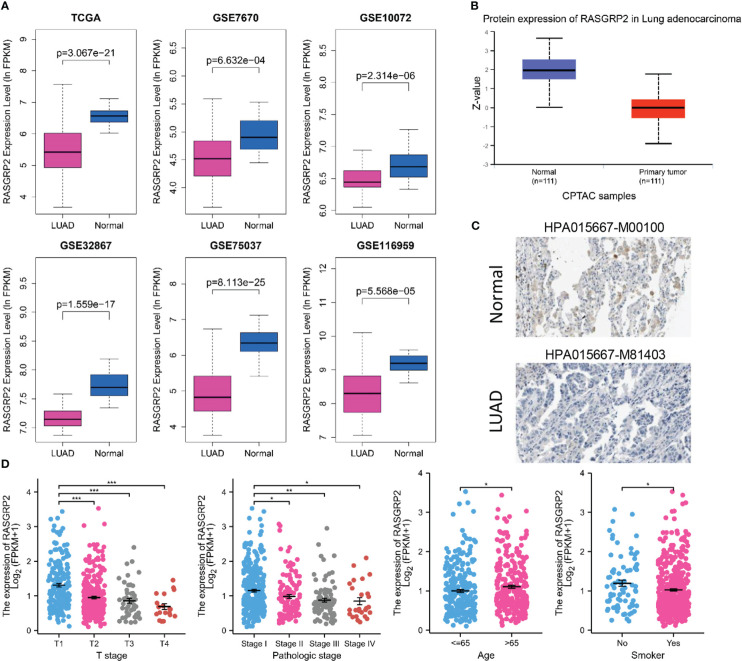
RASGRP2 expression was significantly lower and was associated with malignant clinicopathological parameters in LUAD. **(A)** Data from six different datasets showed that compared with non-tumor tissues, RASGRP2 mRNA was down-regulated in tumor tissues (P < 0.05). **(B)** Protein expression of RASGRP2 in LUAD was lower than that in normal tissues (P < 0.05) according to CPTAC samples. **(C)** Immunohistochemistry demonstrated the lower expression of RASGRP2 in LUAD. **(D)** The expression of RASGRP2 correlated with T stage, pathologic stage, age and smoke. ***P < 0.001, **P < 0.01, *P < 0.05.

To establish the relationship between RASGRP2 and LUAD, we evaluated RASGRP2 expression levels in LUAD tissues and non-tumor tissues through the data from GEO databases. It revealed that both mRNA and protein expression levels of RASGRP2 were significantly reduced in LUAD (P < 0.001) (**Figures 2A, B**). The IHC results from the HPA database also demonstrated the low expression of RASGRP2 in LUAD (**Figure 2C**). Additionally, lower expression of RASGRP2 was also correlated with advanced T classification (P < 0.001) and pathologic stage (P < 0.05), age (P < 0.05) and smoke (P < 0.05) (**Figure 2D**).

**Figure 3:**
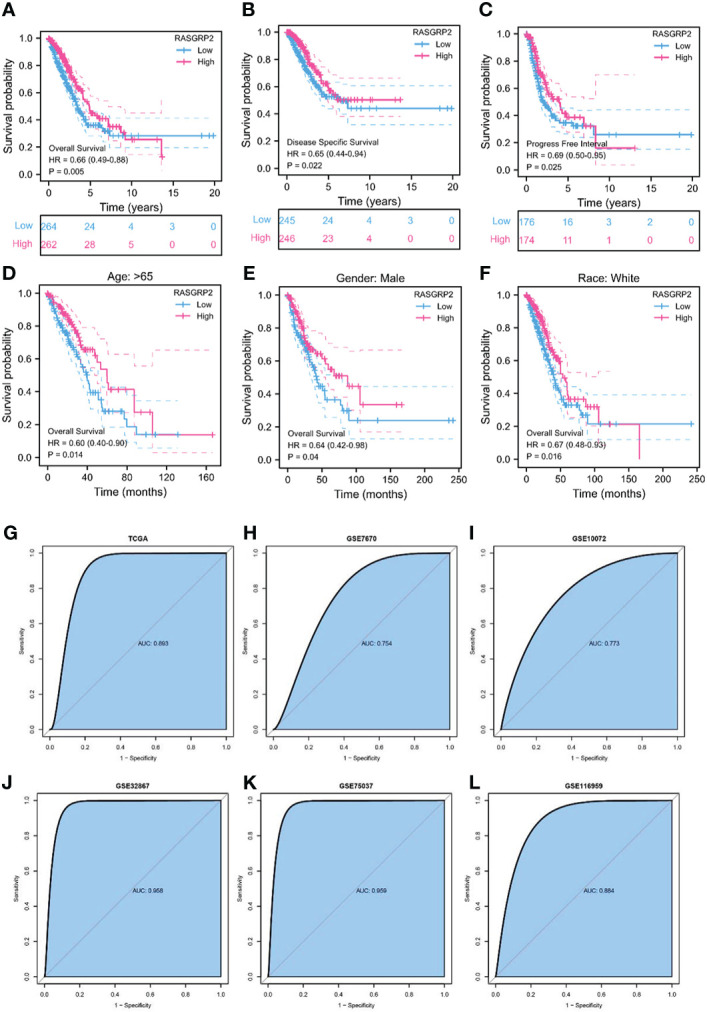
The prognosis and diagnostic value of RASGRP2 in LUAD. The OS **(A)**, DSS **(B)**, and PFI **(C)** survival curves analysis indicated that patients with lower RASGRP2 had poor prognosis (P < 0.05). **(D-F)** Subgroup analysis for age greater than 65 years, male and white patients. **(G–L)** The diagnostic ROC curves of RASGRP2 in TCGA, GSE7670, GSE10072, GSE32867, GSE75037 and GSE116959 datasets.

To further assess the prognostic and diagnostic values of RASGRP2, Kaplan-Meier curves compared overall survival (OS) (P = 0.005), disease-specific survival (DSS) (P = 0.022) and progression-free interval (PFI) (P = 0.025) between patients with high and low expression of RASGRP2 (**Figures 3A–C**) in TCGA-LUAD cohort. Subgroup analysis showed that a low RASGRP2 expression was significantly correlated with poor prognosis in while (P = 0.016) male patients (P = 0.04) over 65 years old (P = 0.014) (**Figures 3D–F**). **Table 1** suggested that RASGRP2 is an independent prognostic factor for LUAD patients. Receiver operating characteristic (ROC) curve analyses were performed to prove the diagnostic value. The area under the curve (AUC) of RASGRP2 expression ranged from 0.754 to 0.959, representing the strong correlation between RASGRP2 and LUAD diagnosis (**Figures 3G-L**). Taken together, RASGRP2 might be an underlying biomarker of diagnosis and prognosis in LUAD.

**Table 1:** Univariate and Multivariate Cox regression analysis of OS in TCGA-LUAD.

Clinical feature	Group	Univariate analysis		Multivariate analysis	
		Hazard ratio (95% CI)	P value	Hazard ratio (95% CI)	P value
Age	<=65	Reference			
	>65	1.223 (0.916-1.635)	0.172		
Gender	Female	Reference			
	Male	1.070 (0.803-1.426)	0.642		
Smoker	No	Reference			
	Yes	0.894 (0.592-1.348)	0.591		
Pathologic stage	I & II	Reference			
	III & IV	2.664 (1.960-3.621)	**< 0.001**	2.554 (1.874-3.480)	**< 0.001**
RASGRP2	Low	Reference			
	High	0.657 (0.490-0.880)	**0.005**	0.727 (0.541-0.978)	**0.035**

The bold represents P < 0.05.

### 3.3 Mutation, methylation and phosphorylation of RASGRP2

**Figure 4:**
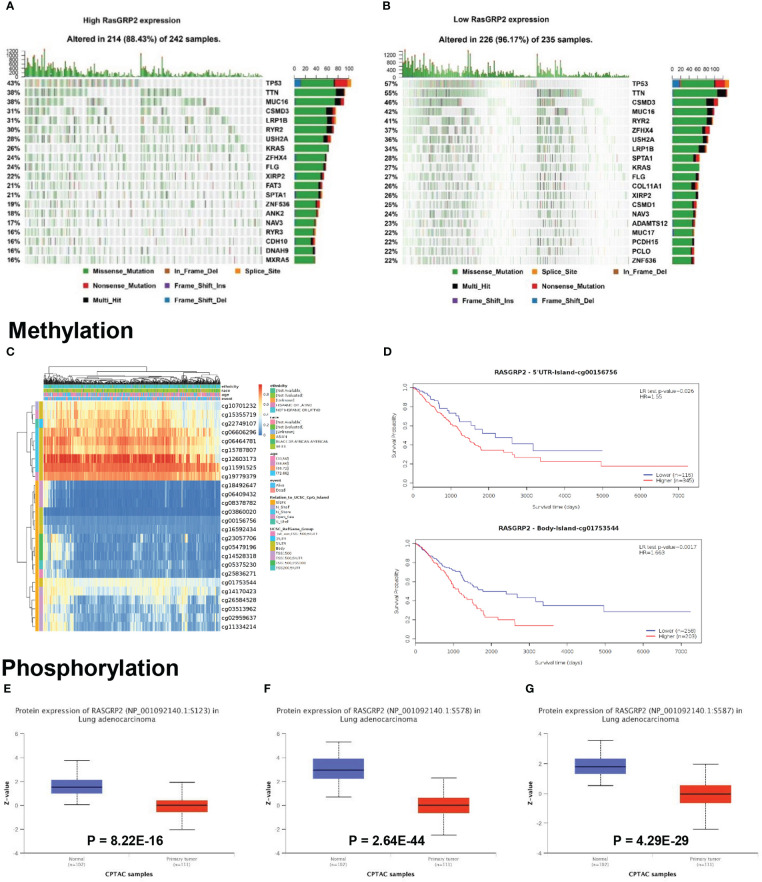
Mutation, methylation and phosphorylation of RASGRP2 in LUAD. **(A, B)** Differential somatic mutations were found in LUAD with high and low RASGRP2 expression. **(C)** Heatmap between the methylation level and RASGRP2 expression. **(D)** Prognostic value of cg00156756 and cg01753544. **(E–G)** The phosphorylation of RASGRP2 at S123, S578, S587 was analyzed in LUAD. The results were obtained from the UALCAN database.

LUAD is a kind of severe cancer with high heterogeneity and genetic factors (15). The association between RASGRP2 expression and somatic mutations was analyzed from TCGA-LUAD. The top 20 significantly different somatic mutations are shown in **Figures 4A, B**. The results showed a high frequency of mutations in TP53, TTN, CSMD3, MUC16 and KRAS in the low RasGRP2 expression group. These mutated genes are known biomarkers of LUAD and are of great value for evaluating the tumor malignant progression or therapeutic response. DNA methylation is indispensable in the study of gene epigenetics (16). DNA methylation levels of RASGRP2 with the prognostic value of each single CpG were investigated using the MethSurv tool (**Figure 4C**). There are 2 CpG sites whose methylation levels were strongly associated with prognosis, namely: cg00156756 and cg01753544 (**Figure 4D**). Next, we compared the phosphorylation of RASGRP2 between normal and tumor tissues using the CPTAC dataset. As summarized in **Figures 4E-G**, we found that the phosphorylation levels of S123, S578 and S587 of RASGRP2 in primary tumor tissues of LUAD were significantly reduced. The most important function of RasGRP2 is the activation of the small GTPase Rap1. In general, phosphorylation of RASGRP2 was regulated by protein kinase A (PKA) and extracellular signal-regulated kinases 1/2 (ERK1/2) and was inhibited to activate the small GTPase Rap1 (17–20).

### 3.4 Construction of the upstream regulatory network of RASGRP2

**Figure 5:**
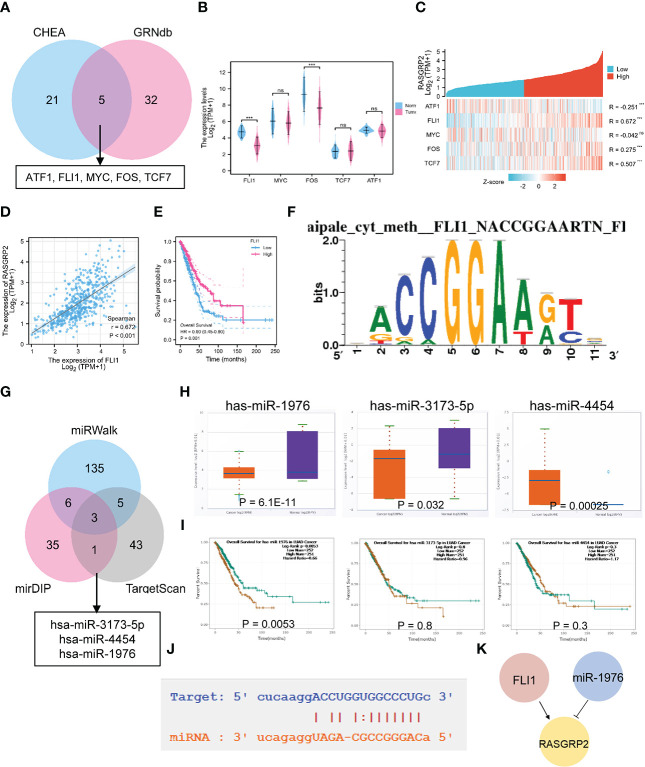
The TF-miRNA-mRNA regulatory network of RASGRP2. **(A)** Venn diagram found that ATF1, FLI1, MYC, FOS and TCF7 were potential transcription factors of RASGRP2. **(B)** The expression levels of ATF1, FLI1, MYC, FOS and TCF in TCGA-LUAD. **(C)** Heatmap showed the correlation of ATF1, FLI1, MYC, FOS and TCF with RASGRP2. **(D, E)** Correlation scatter plot of FLI1 and RASGRP2 and its prognosis curve. **(F)** Predicted TF binding motif. **(G)** Venn diagram found that has-miR-3173-5P, has-miR-4454 and has-miR-1976 were potential miRNAs of RASGRP2. **(H, I)** The expression levels and prognosis curve of has-miR-3173-5P, has-miR-4454 and has-miR-1976. **(J)** Predicted interaction of RASGRP2 and has-miR-1976. **(K)** Potential upstream TF-miRNA-mRNA regulatory network of RASGRP2.

We analyzed the upstream regulation mechanism of RASGRP2, screening transcription factors (TFs) and miRNA, which targeted RASGRP2. We found 26 TFs possibly targeting RASGRP2 from the CHEA database (**Table S3**), 38 from the GRNbd database (**Table S4**). As a result, we screened ATF1, FLI1, MYC, FOS and TCF7 as the most vital TFs (**Figure 5A**). We analyzed the expression levels of the 5 potential TFs and their correlation with RASGRP2 in LUAD (**Figures 5B, C**). The results suggested that FLI1 expression was down-regulated in LUAD and had the highest correlation with RASGRP2 (r = 0.672, P < 0.001) (**Figure 5D**). In addition, LUAD patients with high FLI1 expression had a better prognosis (**Figure 5E**), consistent with the RASGRP2. **Figure 5F** showed the predicted TF binding motif. Subsequently, we explored the upstream miRNA regulator of RASGRP2 based on miRWalk, mirDIP and TargetScan. First, the predicted miRNAs were obtained by the intersection of the three datasets (**Figure 5G**). Next, expression analysis and prognostic analysis were used for further screening. The results indicated that the miRNA most likely to be involved in the post-transcriptional regulation of RASGRP2 was hsa-miR-1976 (**Figures 5H, I**). We also presented the complementary sequences between RASGRP2 and hsa-miR-1976 (**Figure 5J**). Based on these, a FLI1-hsa-miR-1976-RASGRP2 transcriptional network in LUAD was constructed (**Figure 5K**).

### 3.5 The potential mechanisms of RASGRP2 in LUAD

**Figure 6:**
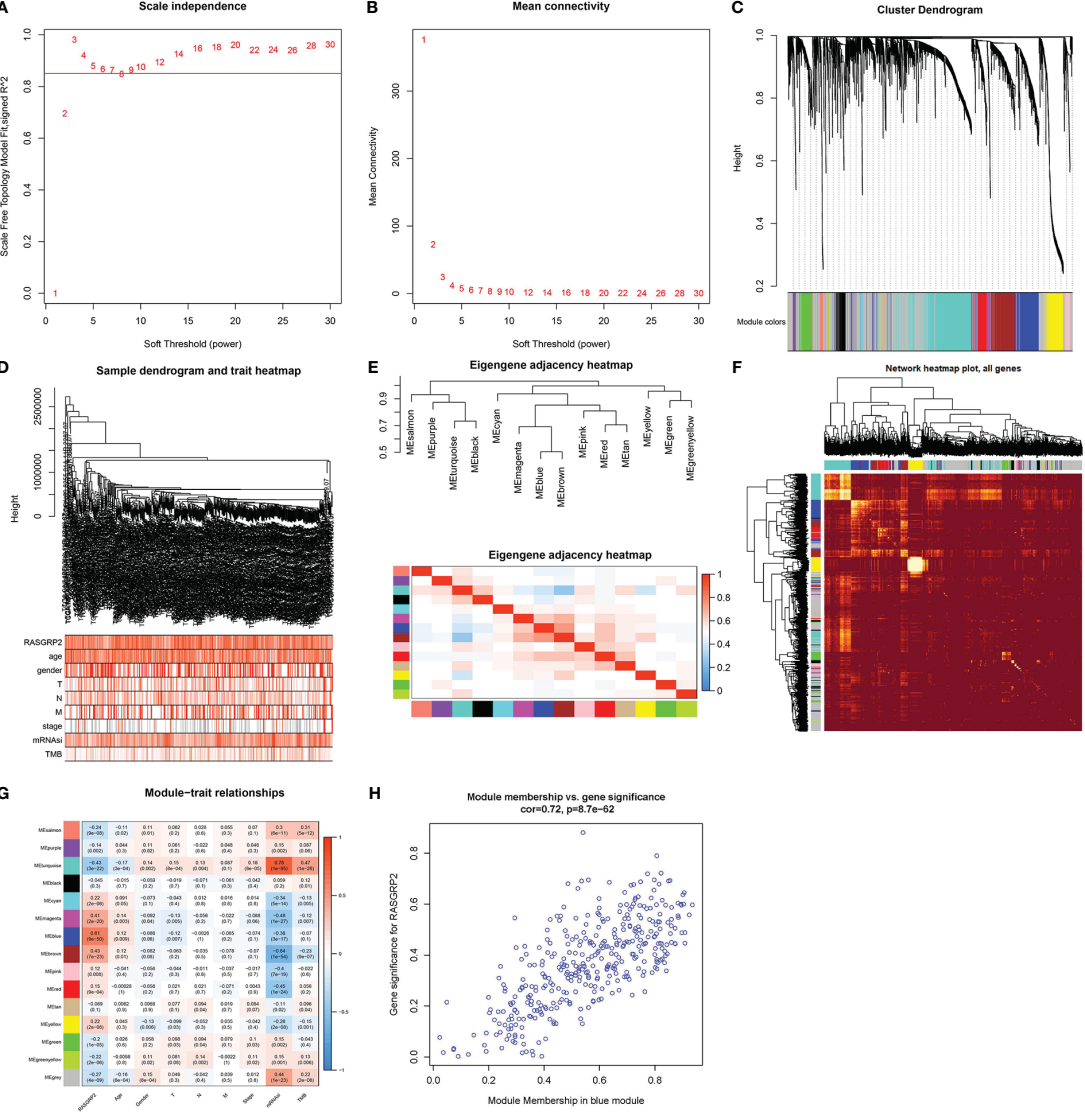
Screening for RASGRP2-related modules and genes in LUAD. **(A, B)** Calculation of the scale-free fit index of various soft-thresholding powers (β) and analysis of the mean connectivity of various soft-thresholding powers (β). **(C–F)** Cluster dendrogram of LUAD patients. According to the dissimilarity measure (1-TOM), 473 were clustered into 15 modules. **(G)** The correlation heatmap between clinicopathological parameters and modules when RASGRP2 was the research object of LUAD. **(H)** Scatter plot of the blue module.

To elucidate the related genes of RASGRP2 and to further explore the potential counteractive of RASGRP2 in LUAD development, we performed weighted gene co-expression network analysis (WGCNA) (**Figures 6A–F**). All the DEGs were grouped into 15 modules by average linkage hierarchical clustering. The blue module, which comprised 379 genes, showed the highest correlation with RASGRP2 expression (r = 0.61, P < 0.0001) (**Figure 6G**). 43 genes in the blue module were chosen as hub genes (**Figure 6H**).

**Figure 7:**
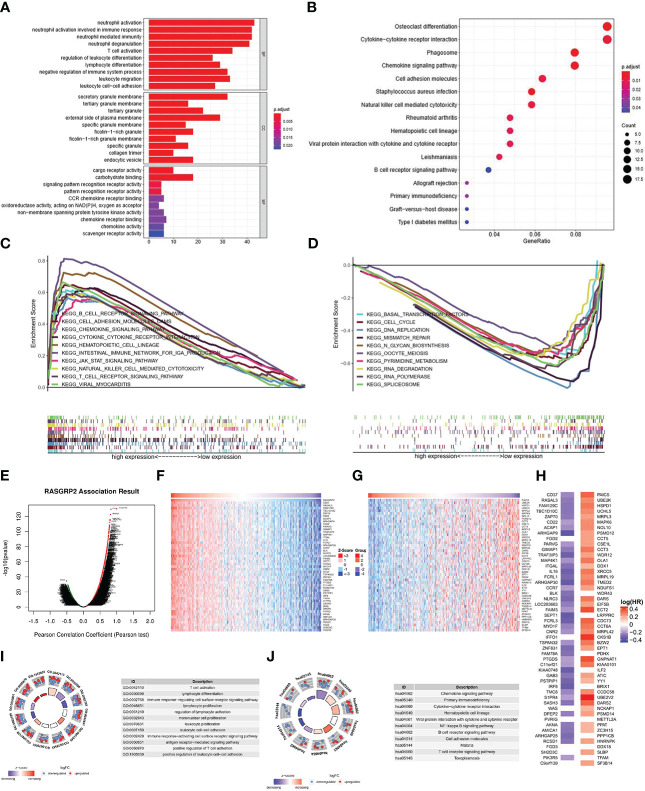
The potential mechanisms of RASGRP2 in LUAD. **(A, B)** GO and KEGG analysis of 397 blue module genes. **(C, D)** GSEA showed the enriched pathways in the high and low RASGRP2 groups. **(E)** The volcano plot showed the whole related genes to RASGRP2. **(F, G)** Top 50 RASGRP2-related genes with positive/negative correlation. **(H)** Survival map of the top 50 genes positively/negatively associated with RASGRP2 in LUAD. **(I)** GO annotations of RASGRP2 in LUAD cohort. **(J)** KEGG analysis of RASGRP2 in LUAD cohort.

According to Gene Ontology (GO) term annotation analysis (**Figure 7A**) of 379 blue module genes, RASGRP2 was associated with neutrophil activation, neutrophil activation involved in immune response and neutrophil-mediated immunity on biological process (BP); secretory granule membrane, tertiary granule and external side of plasma membrane on cellular component (CC); cargo receptor activity and carbohydrate binding on molecular function (MF). Kyoto Encyclopedia of Genes and Genomes (KEGG) analysis (**Figure 7B**) showed RASGRP2 involved in cytokine-cytokine receptor interaction, phagosome, chemokine signaling pathway and cell adhesion. Additionally, Gene Set Enrichment Analysis (GSEA) identified the possible mechanism by comparing the high-RASGRP2 expression group with the low-RASGRP2 expression group (**Figures 7C, D**). It was likely that high-RASGRP2 was enriched in B cell receptor signaling pathway and chemokine signaling pathway. And low-RASGRP2 was correlated to basal transcription factors, DNA replication and mismatch repair.

Another module we constructed to better understand the RASGRP2 co-expression genes in LUAD was the LinkFinder module *via* the LinkedOmics web portal (**Figure 7E**). The heatmaps were used to show the top 50 genes (**Figures 7F, G**). In the first 50 positively correlated genes, 43 genes were having a protective hazard ratio (HR) in LUAD. On the other hand, 41 of 45 negatively correlated genes possessed high HR, having the probability of being high-risk markers (**Figure 7H**). GO analysis suggested that RASGRP2 may be involved in the regulation of T cell activation, lymphocyte differentiation and antigen receptor-mediated signaling pathway (**Figure 7I**). KEGG analysis suggested that RASGRP2 may be involved in the chemokine signaling pathway, primary immunodeficiency pathway and cytokine-cytokine receptor interaction pathway (**Figure 7J**).

### 3.6 RASGRP2 was associated with immune infiltration of LUAD

**Figure 8:**
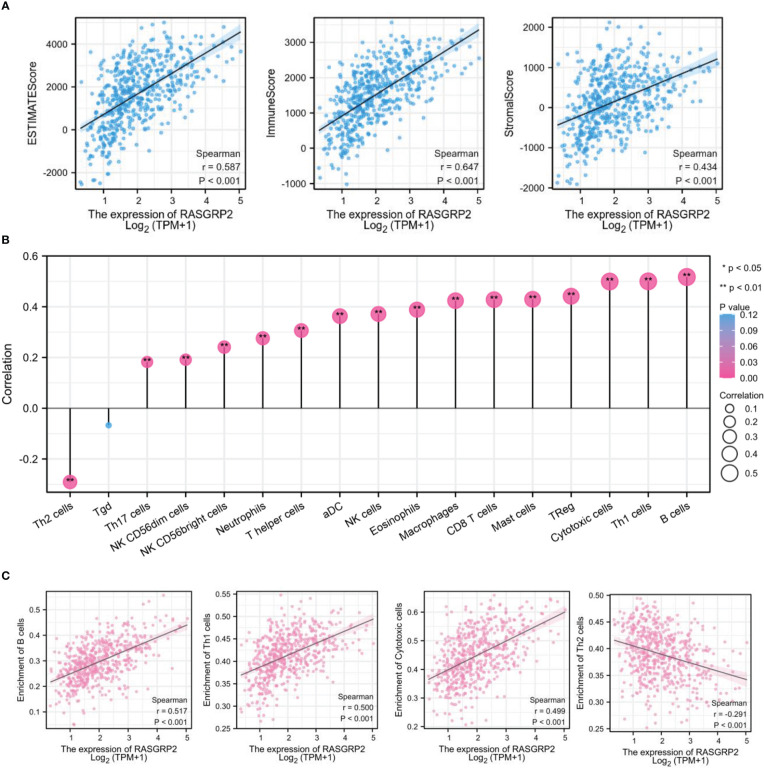
RASGRP2 was positively associated with immune infiltration in LUAD. **(A)** Based on the ESTIMATE, RASGRP2 expression was positively correlated with the ESTIMATE Score, Immune Score and Stromal Score. **(B)** The lollipop plot suggested the correlation of 26 different immune cell subtypes with RASGRP2 expression level. **(C)** The expression of RASGRP2 was positively correlated with B cells, Th1 cells and cytotoxic cells infiltration, but negatively correlated with Th2 cells. **P < 0.01, *P < 0.05.

With previously mentioned results, we found that RASGRP2 was associated with immune cells, cytokine and chemokine, so we sought to explore the relationship between RASGRP2 and immune infiltration. ESTIMATE algorithm for TCGA-LUAD showed that the expression of RASGRP2 in LUAD had a positive correlation with ESTIMATE Score (r = 0.587, P < 0.001), Immune Score (r = 0.647, P < 0.001) and Stromal Score (r = 0.434, P < 0.001) (**Figure 8A**). The level of RASGRP2 expression was positively correlated with the infiltration of most of the immune cells (**Figure 8B**), the top 3 were B cells (r = 0.517, P < 0.001), Th1 cells (r = 0.500, P < 0.001) and cytotoxic cells (r = 0.499, P < 0.001). However, the expression of RASGRP2 was negatively correlated with Th2 cells (r = -0.291, P < 0.001) (**Figure 8C**).

### 3.7 Single-cell analysis of the expression of RASGRP2 in different immune cells of LUAD

**Figure 9:**
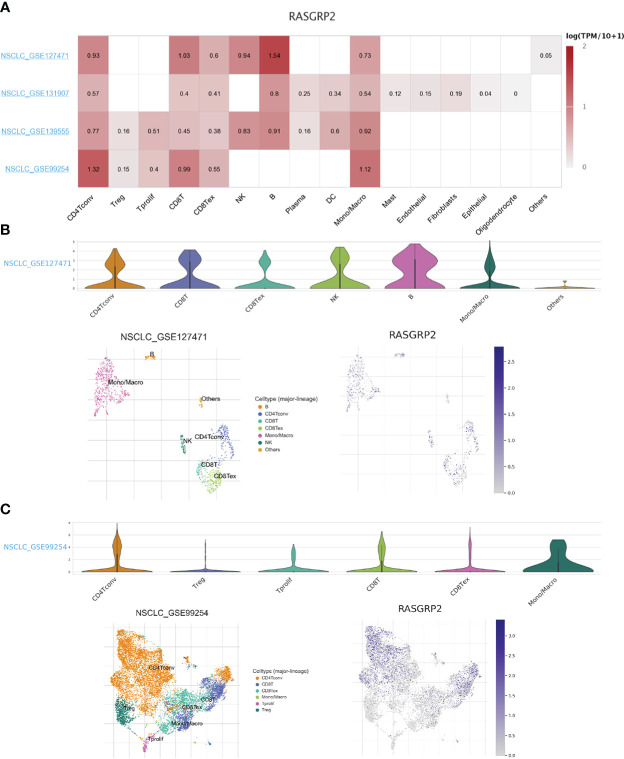
Single-cell analysis of the expression of RASGRP2 in different immune cells of LUAD. **(A)** Heatmap of the correlation of RASGRP2 with immune cell infiltration levels in 4 independent datasets based on the scRNA-seq database. **(B, C)** Violin plot and single-cell atlas of NMUR1 and immune cell infiltration in NSCLC_GSE127471 and NSCLC_GSE99254.

Based on the scRNA-seq TISCH database, we obtained 4 independent datasets of LUAD (NSCLC_GSE127471, NSCLC_GSE131907, NSCLC_GSE139555 and NSCLC_GSE99254) for single-cell sequencing to explore the correlation of immune cell distribution with RASGRP2 expression levels at the single-cell level (**Figure 9A**). In the NSCLC_GSE127471 dataset, higher levels of RASGRP2 expression were found in CD8 T cells, NK cells and B cells (**Figures 9A, B**). Furthermore, RASGRP2 also showed a trend of high expression in CD4 T cells and monocyte-macrophages in the NSCLC_GSE99254 dataset (**Figures 9A, C**). This may be due to the heterogeneity of the tumors that caused the differences between the datasets. Taken together, RASGRP2 expression levels were significantly correlated with immune cell types and their proportions, which may influence immunotherapy response.

### 3.8 Analysis of the correlation between RASGRP2 and immunoinhibitors, immunostimulators, chemokines and chemokine receptors

**Figure 10:**
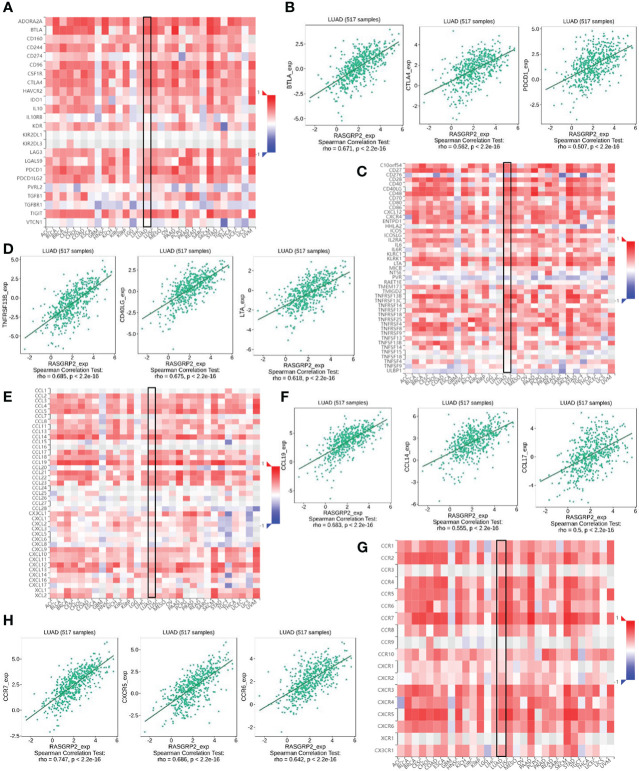
The correlation between RASGRP2 and immunoinhibitors, immunostimulators, chemokines and chemokine receptors *via* TISIDB analysis. **(A, B)** The RASGRP2 expression was positively associated with most of the immunoinhibitors. **(C, D)** The RASGRP2 expression was positively associated with most of the immunostimulators. **(E, F)** The RASGRP2 expression was positively associated with most of the chemokines. **(G, H)** The RASGRP2 expression was positively associated with most of the chemokine receptors.

Then we further verified the correlation between RASGRP2 and immunoinhibitors, immunostimulators, chemokines and chemokine receptors *via* TISIDB analysis. Our results suggested that the RASGRP2 expression was positively associated with most of the immunoinhibitors (**Figure 10A**). As for LUAD, the top 3 were BTLA (rho = 0.671, P < 2.2e-16), CTLA4 (rho = 0.562, P < 2.2e-16) and PDCD1 (rho = 0.507, P < 2.2e-16) (**Figure 10B**). RASGRP2 expression was positively associated with most of the immunostimulators (**Figure 10C**). As for LUAD, the top 3 were TNFRSF13B (rho = 0.685, P < 2.2e-16), CD40LG (rho = 0.675, P < 2.2e-16) and LTA (rho = 0.618, P < 2.2e-16) (**Figure 10D**). RASGRP2 expression was positively associated with most of the chemokines (**Figure 10E**). As for LUAD, the top 3 were CCL19 (rho = 0.683, P < 2.2e-16), CCL14 (rho = 0.555, P < 2.2e-16) and CCL17 (rho = 0.500, P < 2.2e-16) (**Figure 10F**). RASGRP2 expression was positively associated with most of the chemokine receptors (**Figure 10G**). As for LUAD, the top 3 were CCR7 (rho = 0.747, P < 2.2e-16), CXCR5 (rho = 0.686, P < 2.2e-16) and CCR6 (rho = 0.642, P < 2.2e-16) (**Figure 10H**).

### 3.9 Validation of the expression level of RASGRP2 and the correlation with immune-related markers based on a real-world cohort

**Figure 11:**
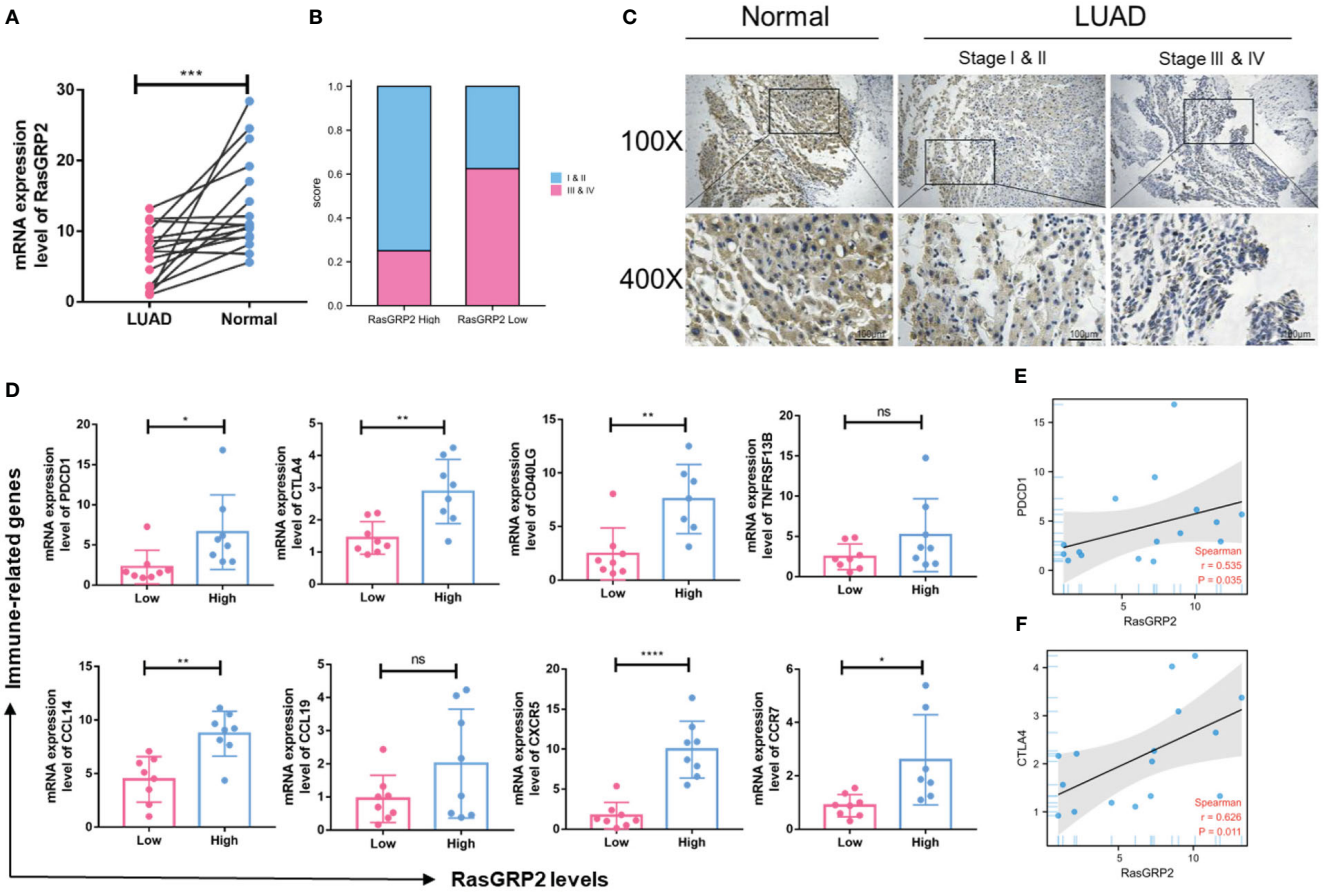
Validation of the expression level of RASGRP2 and immune-related molecular infiltration in a real-world cohort. **(A)** mRNA expression levels of RASGRP2. **(B, C)** The expression level of RASGRP2 correlated with the pathological stages. **(D)** The expression levels of PDCD1, CTLA4, CD40LG, CCL14, CXCR5 and CCR7 were higher in the high-RASGRP2 expression group in the real-world cohort. **(E, F)** RASGRP2 was positively correlated with PDCD1 and CTLA4 in the real-world cohort. ****P < 0.0001, ***P < 0.001, **P < 0.01, *P < 0.05, ns, no significance.

To validate the results mentioned above, we analyzed the RASGRP2 expression level in 16 paired LUAD tissues and adjacent tissues by qRT-PCR (**Figure 11A**). In addition, patients with advanced stage had lower RASGRP2 expression levels (**Figures 11B, C**). We also probed the correlation between RASGRP2 and immune-related molecules that were strongly correlated with RASGRP2 in TCGA-LUAD. The expression levels of PDCD1, CTLA4, CD40LG, CCL14, CXCR5 and CCR7 were higher in the high-RASGRP2 expression group. But TNFRSF13B and CCL19 did not show a statistically significant correlation (**Figure 11D**). The reason might be the limited samples. As popular immune checkpoints, PDCD1 and CTLA4 are playing an increasingly extensive role in clinical practice. We analyzed the correlation between RASGRP2 and PDCD1/CTLA4. **Figures 11E, F** showed that RASGRP2 was positively correlated with PDCD1 (r = 0.535, P = 0.035) and CTLA4 (r = 0.626, P = 0.011). Subsequently, we further verified the relationship between RASGRP2 and PDL1 in cell lines double staining. It is well known that PDL1 is considered to be a predictor of the effectiveness of immunotherapy (21). The immunofluorescence experiment confirmed that high RASGRP2 corresponded to high PDL1 (**Figure S2**). The above results suggested that patients with high RASGRP2 levels might benefit more from immunotherapy.

### 3.10 RASGRP2 inhibited cell proliferation by regulating apoptosis in LUAD

**Figure 12:**
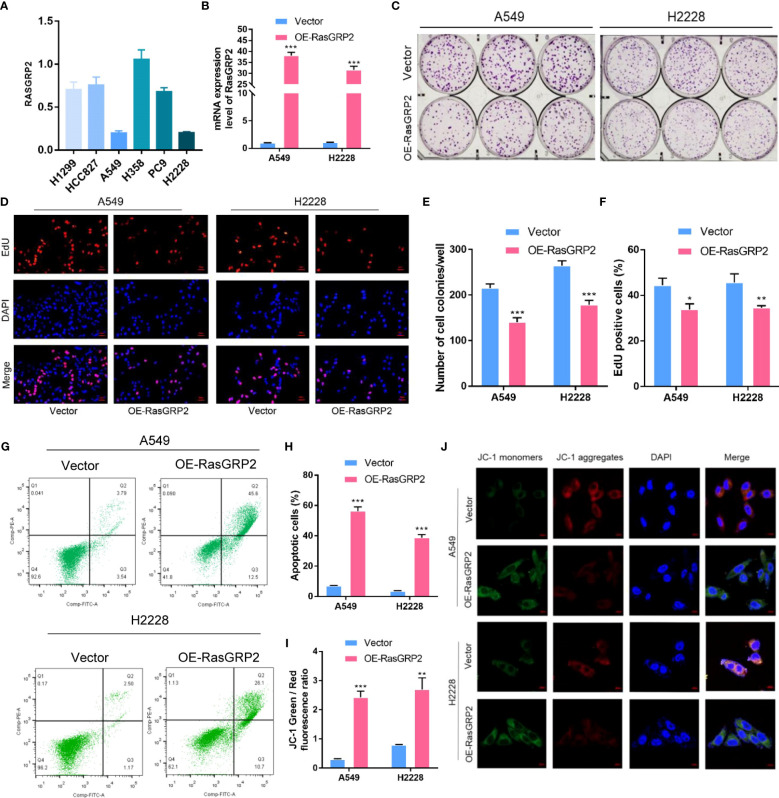
Overexpression of RASGRP2 inhibited the proliferation by regulating apoptosis in LUAD. **(A)** The level of RASGRP2 expression in 6 lung cancer cell lines. **(B)** RASGRP2 overexpression efficiency was verified in A549 and H2228 cell lines. **(C–F)** Clone formation assay and EdU assay suggested that high RASGRP2 inhibited cell proliferation. **(G, H)** Flow cytometry analysis suggested that overexpression of RASGRP2 promoted apoptosis. **(I, J)** JC-1 staining suggested that overexpression of RASGRP2 was associated with decreased mitochondrial membrane potential. *** P < 0.001, ** P < 0.01, * P < 0.05.

First, we analyzed the expression levels of RASGRP2 in 6 different lung cancer cell lines (22). RASGRP2 was lower expressed in A549 and H2228, but higher expressed in H358 (**Figure 12A**). Next, we overexpressed RASGRP2 in A549 and H2228 cell lines and performed efficiency validation (**Figure 12B**). The results of the colony formation assay and EdU assay confirmed that high RASGRP2 significantly impaired the proliferation ability (**Figures 12C–F**). Flow cytometry analysis showed that overexpression of RASGRP2 increased the proportion of apoptotic cells (**Figures 12G, H**). The process of apoptosis is often accompanied by the destruction of MMP. The increased JC-1 monomers ratio suggested the occurrence of apoptosis when RASGRP2 was overexpressed (**Figures 12I, J**). Meanwhile, we obtained the reverse results by silencing RASGRP2 in H358 (**Figure S3**).

## 4 Discussion

In this study, 7 GEFs were identified as the most valuable candidates, namely MMP1, DOCK4, CFTR, RASGRP2, KLF4, MIF and ECT2. After rigorous data retrieval, we found that it was necessary to establish a comprehensive landscape illustrating the role of RASGRP2 in the malignant progression and immune regulation of LUAD.

RASGRP is divided into four isoforms, RASGRP1-4. Among them, RasGRP1 and RasGRP4 are suggested to affect T cell development (23–26). Besides, RasGRP1 and RasGRP3 are thought to modulate Ras- ERK signaling cascade, actin polymerization, and further regulate T cell development and T cell progenitors chemotaxis after CCR9 stimulation (27). RasGRP4 is expressed in fibroblast-like synoviocytes (FLS) in rheumatoid arthritis (RA) patients, elevating the FLS proliferation and MMP-1 production (28). RASGRP2 is the most divergent member of the RASGRP family. Unlike RasGRP1, 3, and 4, RASGRP2 is mainly present in the cytoplasm and translocates to the cell membrane once stimulated. It specifically activates Rap1 and Rap2, but lacks Ras exchange activity *in vivo* (29, 30). In addition, RASGRP2 is the only Rap1-GEF active in platelets and plays a central role in platelet activation. It is considered a new target for antiplatelet therapy (31). Jun-Ichi Takino reported that RASGRP2 regulated angiogenesis and vascular permeability as a protective factor that maintained vascular endothelial cell homeostasis (32–34).

Here, we focus on RASGRP2 to explore its role in LUAD. We found out that the RASGRP2 expression level was lower in LUAD tissues compared with that in non-tumor tissues by bioinformatics analysis and experimental validation. The decreased expression level of RASGRP2 was associated with advanced stage and poor prognosis. These results indicated that the absence of RASGRP2 might be connected with the development of LUAD. Our lab experiments also confirmed the anti-growth ability and pro-apoptosis ability of RASGRP2.

At the genetic level, cancer is a clonal process in which somatic gene mutations accumulate to a certain extent, resulting in abnormal growth of normal cells. Studies have demonstrated that local DNA hypermethylation can be used as an auxiliary marker to clinically diagnose cancer and predict prognosis (35–37). For example, the glutathione-S-transferase P1 (GSTP1) gene is hypermethylated in 70-80% of prostate cancer patients, but not in normal hyperplastic prostate tissues (37). In this study, we identified 2 CpG sites of RASGRP2 which were strongly associated with prognosis. Since DNA methylation is reversible, hypermethylated genes can be a target for drug therapy in cancer.

More recently, the spread and efficacy of targeted immunotherapy have started to change cancer management (38, 39). Given the complex relationship between the tumor immune microenvironment and host immunity, predictive biomarkers are needed for personalized therapy. Further analysis found that the expression of RASGRP2 was positively correlated with T helper cells and Th1 cell infiltration, but negatively correlated with Th2 cells. It is well known that dysregulation of T cell subsets is common in cancer patients (40). Th1/Th2 imbalance not only affects the infiltration of other immune cells, but is also associated with complications after radiotherapy (41). Our results suggested that the expression of RASGRP2 may cause Th1/Th2 shift. The intervention of RASGRP2 expression at the gene level may provide a new target for the immunotherapy of LUAD. Besides, RASGRP2 was positively linked to some immune molecules, such as PDCD1, CDLA4, CD40LG, CCL14, CXCR5 and CCR7. Currently, dual immune checkpoint inhibitors with anti-PD-1/PD-L1 and anti-CTLA4 monoclonal antibodies are being widely evaluated for tumor therapy. Nivolumab (IgG4 anti-PD-1) plus ipilimumab (fully humanized IgG1 anti-CTLA4) is one of the most popular combined immunotherapy regimens (42, 43). Real-world cohort results showed that the expression level of RASGRP2 was positively correlated with PDCD1 and CTLA4. It seemed that high RASGRP2 broke the immunosuppressive barrier and might be a biomarker for a good response to immunotherapy. However, the results from our real-world cohort could not verify the connection between RASGRP2 and TNFRSF13B and CCL19. The reason might be a result of the limited samples and tumor heterogeneity.

Unlike traditional immune checkpoint inhibitors, the development of immunostimulators to reactivate the immune system is undoubtedly a new therapeutic idea. It has been reported the immunostimulators mainly focus on the tumor necrosis factor receptor (TNFR) family and B7-CD28 family. Currently, a number of agonistic antibodies have entered the clinical stage (44–46). TNFRSF4, also known as OX40, activates the PI3K/PKB, NF-κB1 and NFAT pathways and enhances T cell-mediated antitumor immunity, thereby improving survival in several preclinical cancer models, including B16 melanoma and lung cancer (47).

In order to further explore the mechanism of the regulation of RASGRP2 in malignant progression and immune infiltration, combined with the GSEA results, we found a potential pathway: JAK3-STAT5, which may be related to RASGRP2. We analyzed the association of the JAK family (JAK1-3 and TYK2) and the STAT family (STAT1-6) with RASGRP2 in LUAD (**Figure S4A**). The JAK3- STAT5 signaling pathway has the strongest positive correlation with RASGRP2 (**Figure S4B**). Subsequently, western blot analysis showed that overexpression of RASGRP2 increased the phosphorylation levels of JAK3 and STAT5 (**Figure S4C**). Many studies have reported that activation of the JAK-STAT pathway promotes cell apoptosis (48–50), which is consistent with our results. However, depending on the response signal, the JAK-STAT pathway plays a dual role of “anti-tumor” and “pro-tumor” in the tumor immune microenvironment (51–53). Therefore, large-scale standardized animal experiments and clinical trials are necessary, and our team is working on that.

Although our study had many interesting findings, there are still some limitations in this study. Firstly, the mechanisms of action of RASGRP2 in LUAD are necessary to be validated *in vivo* and *in vitro*. Secondly, immunotherapy cohorts and single-cell analysis need to be performed to correct for systematic biases in different databases. This study focuses on bioinformatics analysis and preliminary functional exploration to screen out potential biomarkers. We will implement these limitations in future studies.

## 5 Conclusion

RASGRP2 was a potential immune-related biomarker of LUAD. In addition, RASGRP2 was involved in the malignant progression of LUAD through the regulation of mitochondrial-dependent apoptosis.

## Data availability statement

All original materials in the study are included in the article/supplementary material, further inquiries can be directed to the corresponding authors.

## Ethics statement

The studies involving human participants were reviewed and approved by the Ethics Committees of the Xiangya Hospital. The patients/participants provided their written informed consent to participate in this study.

## Author contributions

YL and YO designed and drafted the manuscript. ZF, ZJ, and JM analyzed the data. XZ and CC performed the experiments. YH and SZ collected the clinical samples. SL and HS supervised the study. All authors contributed to the article and approved the submitted version.

## Supplementary material

Supplementary Figure 1 The clinical correlation analysis of the 7 valuable GEFs. **(A, B)** The expression difference of 7 valuable GEFs between different T stage and pathological stage. **(C)** time-ROC of RASGRP2 in 1-, 2- and 3 years.

Supplementary Figure 2 Analysis of the difference of target proteins under high/low RASGRP2 **(A, B)** The bar charts showed that there was no statistical difference in RASGRP2 expression in patients with KRAS mutant/wild and EML4-Alk positive/negative. **(C, D)** The immunofluorescence experiment confirmed that high RASGRP2 corresponded to high PDL1.

Supplementary Figure 3 Knockdown of RASGRP2 promoted the proliferation by regulating apoptosis in LUAD. **(A)** RASGRP2 silencing efficiency was verified by qPCR. **(B–E)** Clone formation assay and EdU assay suggested that low RASGRP2 promoted cell proliferation. **(F–I)** Flow cytometry analysis and JC-1 staining suggested that decreased of RASGRP2 inhibited mitochondrial-dependent apoptosis.

Supplementary Figure 4 RASGRP2 may play a role in LUAD *via* the JAK3-STAT5 signaling pathway. **(A, B)** Correlation analysis of RASGRP2 and JAK-STAT pathways. **(C)** Overexpression of RASGRP2 promoted activation of the JAK3-STAT5 signaling pathway.

Supplementary Table 1 Clinicopathological parameters of real-world LUAD cohort from Xiangya Hospital.

Supplementary Table 2 Guanine nucleotide exchange factors (GEFs) from GeneCards.

Supplementary Table 3 Predictive transcription factor in CHEA.

Supplementary Table 4 Predictive transcription factor in GRNdb.
