# Supplementary figures and images for: RASGRP2 is a potential immune-related biomarker and regulates mitochondrial-dependent apoptosis in lung adenocarcinoma
Source: Front Immunol. 2023 Feb 3;14:1100231. doi: 10.3389/fimmu.2023.1100231 (PMC9936229; doi:10.3389/fimmu.2023.1100231)

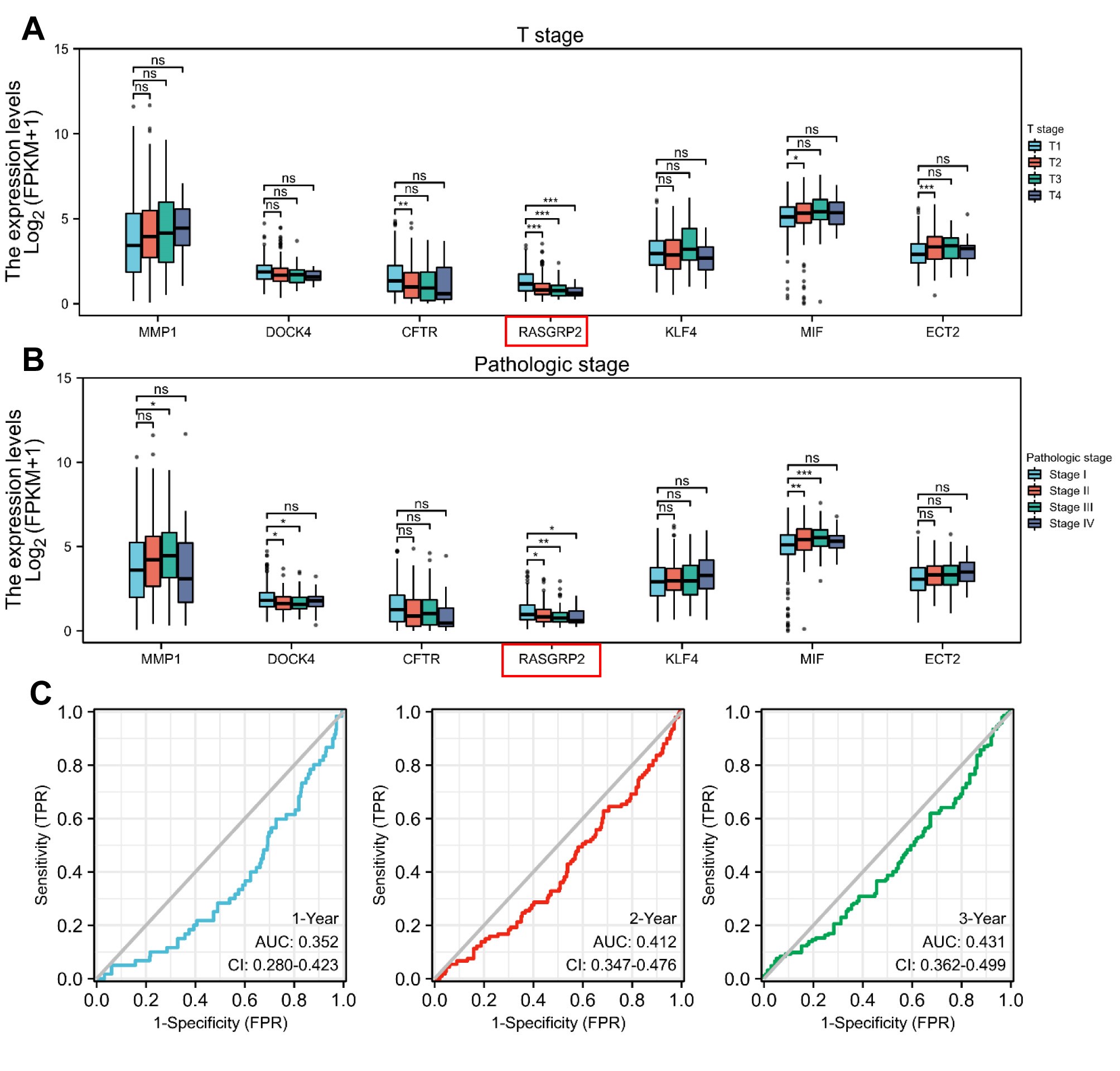

Supplement: Supplementary Figure 1 — The clinical correlation analysis of the 7 valuable GEFs. (A, B) The expression difference of 7 valuable GEFs between different T stage and pathological stage. (C) time-ROC of RASGRP2 in 1-, 2- and 3 years. [file Image_1.jpeg]

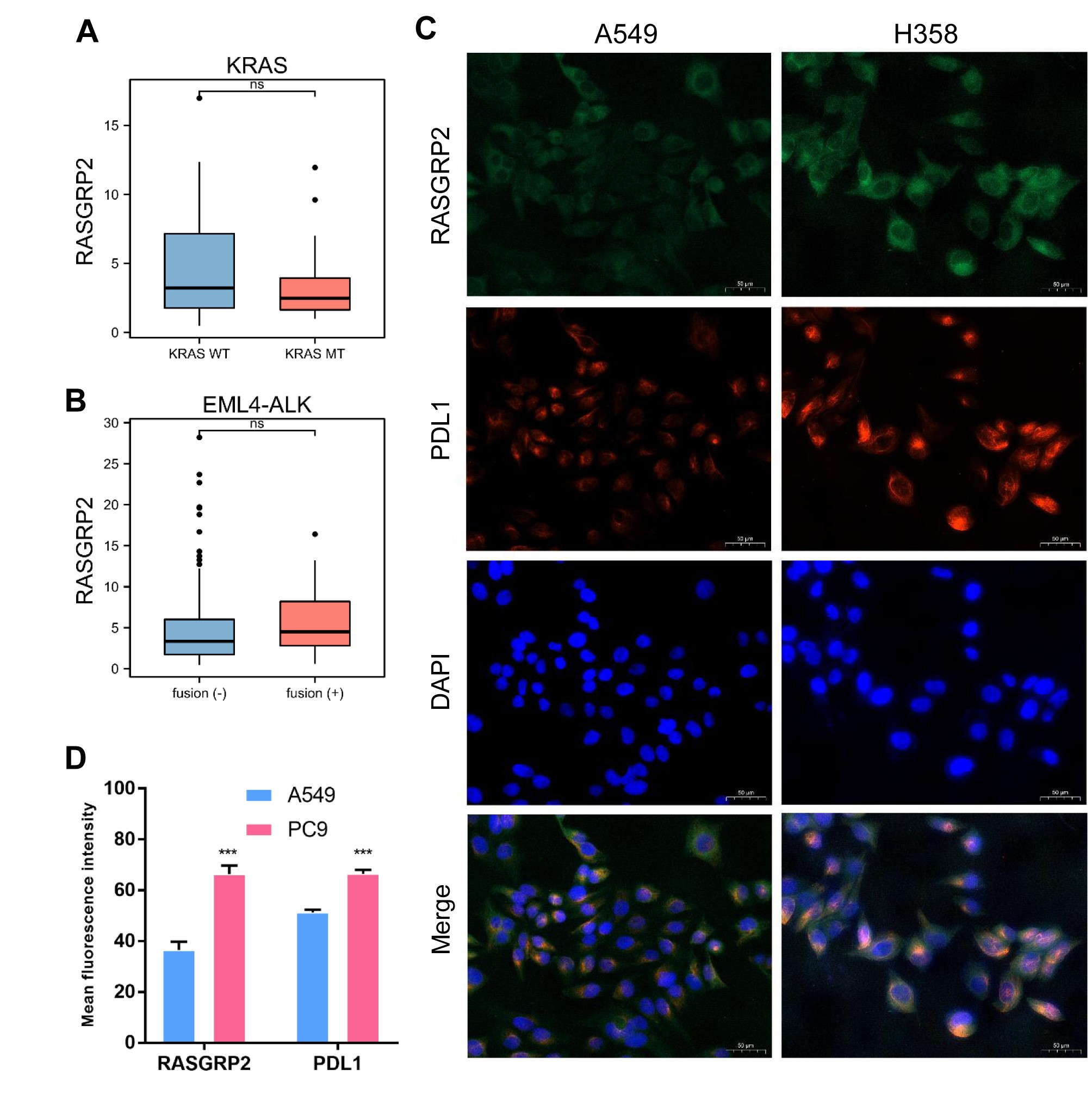

Supplement: Supplementary Figure 2 — Analysis of the difference of target proteins under high/low RASGRP2 (A, B) The bar charts showed that there was no statistical difference in RASGRP2 expression in patients with KRAS mutant/wild and EML4-Alk positive/negative. (C, D) The immunofluorescence experiment confirmed that high RASGRP2 corresponded to high PDL1. [file Image_2.jpeg]

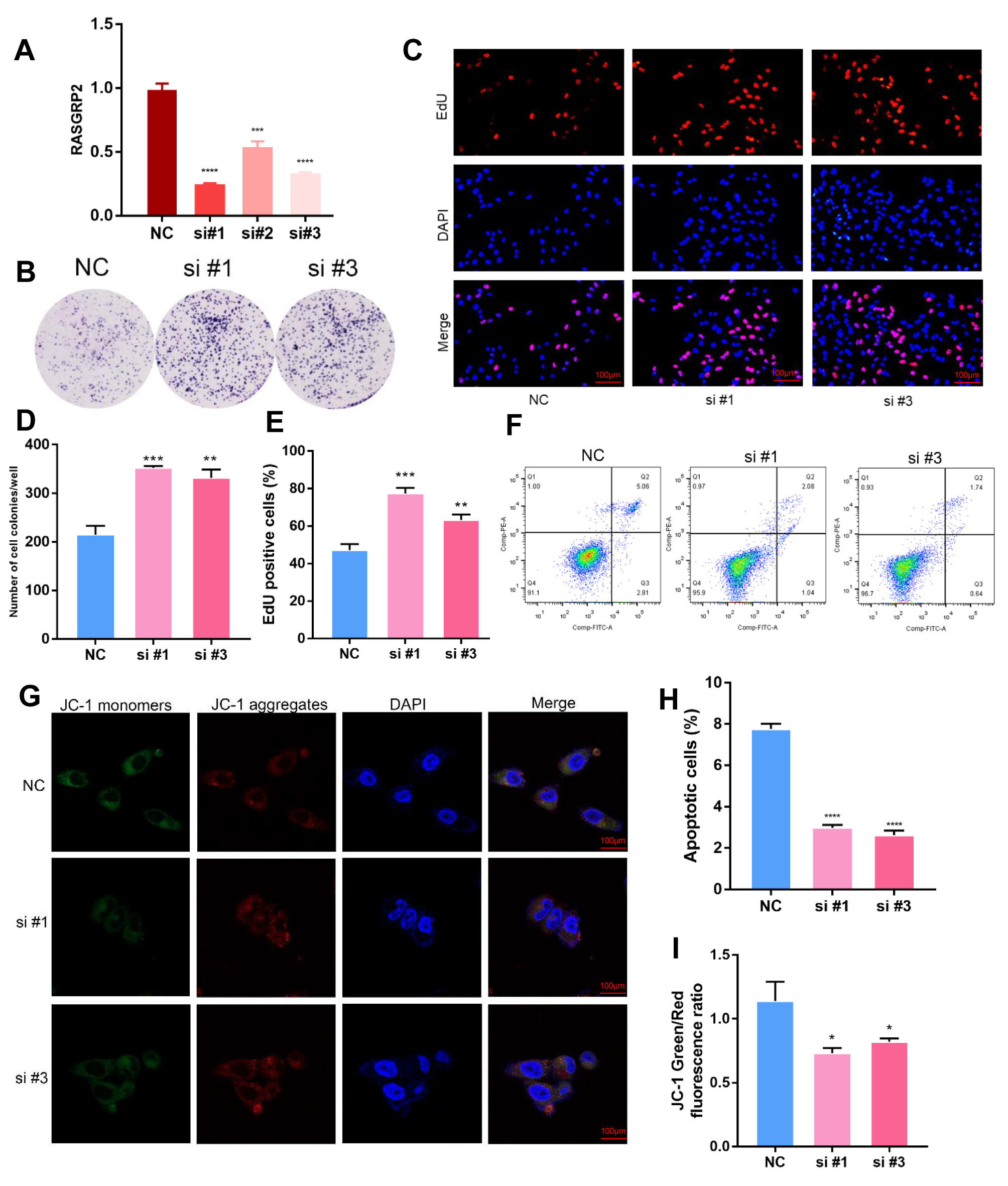

Supplement: Supplementary Figure 3 — Knockdown of RASGRP2 promoted the proliferation by regulating apoptosis in LUAD. (A) RASGRP2 silencing efficiency was verified by qPCR. (B–E) Clone formation assay and EdU assay suggested that low RASGRP2 promoted cell proliferation. (F–I) Flow cytometry analysis and JC-1 staining suggested that decreased of RASGRP2 inhibited mitochondrial-dependent apoptosis. [file Image_3.jpeg]

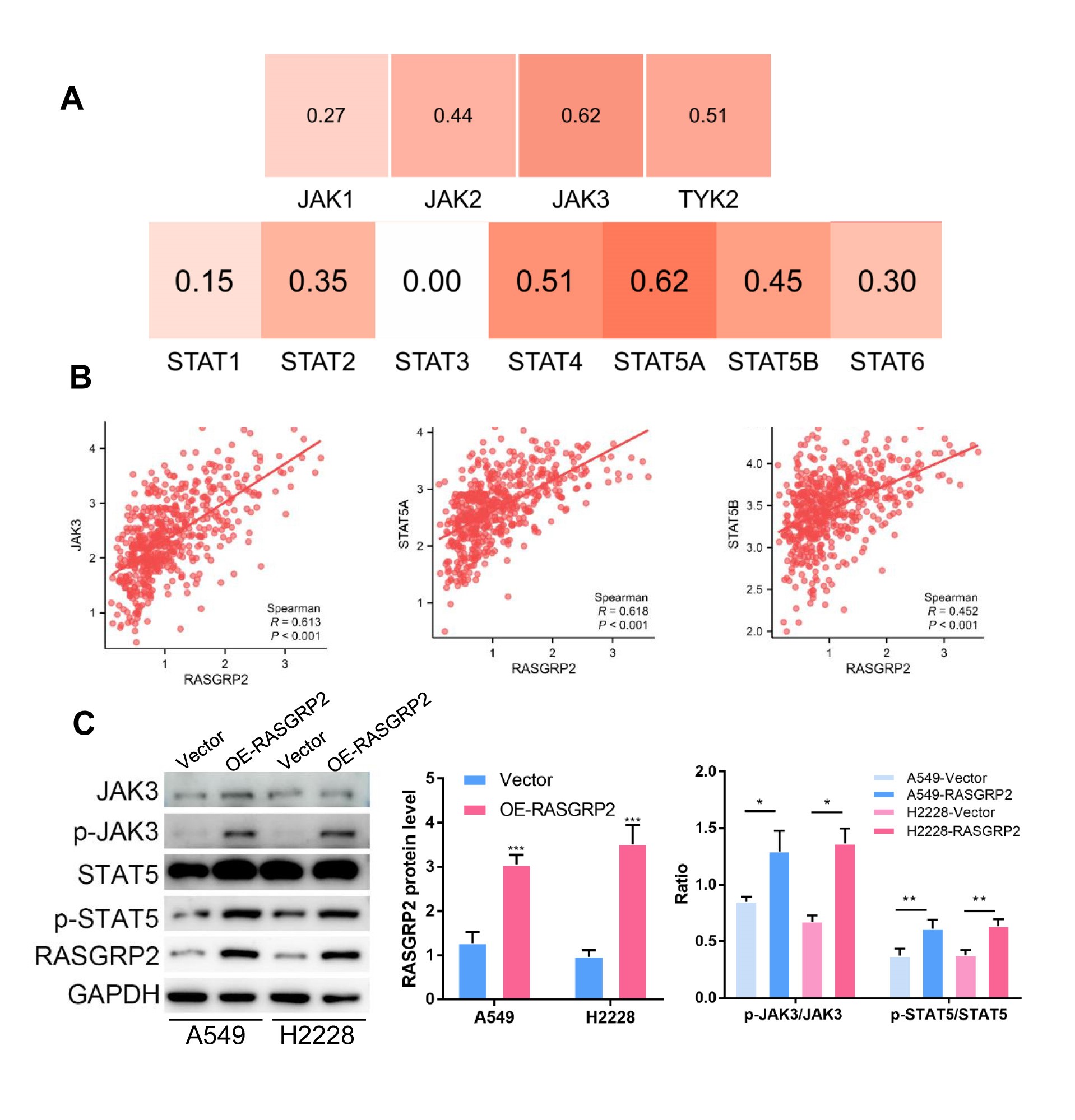

Supplement: Supplementary Figure 4 — RASGRP2 may play a role in LUAD via the JAK3-STAT5 signaling pathway. (A, B) Correlation analysis of RASGRP2 and JAK-STAT pathways. (C) Overexpression of RASGRP2 promoted activation of the JAK3-STAT5 signaling pathway. [file Image_4.jpeg]
