# Supplementary material for: RASGRP2 is a potential immune-related biomarker and regulates mitochondrial-dependent apoptosis in lung adenocarcinoma
Source: Front Immunol. 2023 Feb 3;14:1100231. doi: 10.3389/fimmu.2023.1100231 (PMC9936229; doi:10.3389/fimmu.2023.1100231)
Supplement: Supplementary Table 1 — Clinicopathological parameters of real-world LUAD cohort from Xiangya Hospital. [file Table_1.docx]

| **Patients**  **Table S1. Clinicopathological parameters of real-world LUAD cohort from Xiangya Hospital**  **No.** | **Gender** | **Age (years)** | **Smoke** | **Location** | **Differentiation** | **Stage** | **RasGRP2 relative expression** |
| --- | --- | --- | --- | --- | --- | --- | --- |
| 1 | Male | 55 | Yes | Left upper | Moderately-poorly | Ⅱ | 8.999 |
| 2 | Male | 66 | / | Left Lower | poorly | Ⅲ | 2.075 |
| 3 | Female | 41 | No | Left Lower | well | Ⅰ | 13.217 |
| 4 | Male | 67 | Yes | Right upper | well | Ⅲ | 2.221 |
| 5 | Male | 65 | Yes | Left upper | Moderately | III | 7.159 |
| 6 | Male | 60 | No | Right upper | Moderately | Ⅰ | 11.766 |
| 7 | Female | 59 | No | Right upper/lower | Moderately | Ⅰ | 7.247 |
| 8 | Male | 58 | No | Left upper | Well-moderately | Ⅲ | 4.547 |
| 9 | Male | 53 | Yes | Left upper | Moderately | Ⅱ | 11.490 |
| 10 | Male | 69 | No | Left upper | poorly | Ⅰ | 10.113 |
| 11 | Male | 63 | / | Left upper | / | Ⅱ | 7.382 |
| 12 | Male | 36 | Yes | Right upper | Well-moderately | Ⅱ | 8.577 |
| 13 | Female | 45 | Yes | Left Lower | Moderately | Ⅳ | 1.015 |
| 14 | Male | 61 | Yes | Right upper | Moderately | Ⅲ | 1.307 |
| 15 | Male | 53 | Yes | Right lower | Moderately | Ⅳ | 1.000 |
| 16 | Male | 53 | Yes | Right lower | / | III | 6.130 |
